# Secondary metabolites of *Diaporthe cameroonensis*, isolated from the Cameroonian medicinal plant *Trema guineensis*

**DOI:** 10.3762/bjoc.19.112

**Published:** 2023-10-13

**Authors:** Bel Youssouf G Mountessou, Élodie Gisèle M Anoumedem, Blondelle Matio Kemkuignou, Yasmina Marin-Felix, Frank Surup, Marc Stadler, Simeon Fogue Kouam

**Affiliations:** 1 Department of Chemistry, Higher Teacher Training College, University of Yaoundé I, P.O. Box 47, Yaoundé, Cameroon; 2 Department of Microbial Drugs, Helmholtz Centre for Infection Research (HZI) and German Centre for Infection Research (DZIF), Partner Site Hannover/Braunschweig, Inhoffenstrasse 7, 38124 Braunschweig, Germany; 3 Institute of Microbiology, Technische Universität Braunschweig, Spielmannstraße 7, 38106 Braunschweig, Germany

**Keywords:** alternariol, Diaporthe, endophytic fungi, hemiketal polyketide

## Abstract

From a fresh root of *Trema guineensis* (Ulmaceae), endophytic fungi were isolated, among which a taxon belonging to the new species *Diaporthe cameroonensis*. This strain was fermented in shake flask batch cultures and the broth was extracted with ethyl acetate. From the crude extract, a hemiketal polyketide **1**, and an acetylated alternariol **2** were isolated, along with fifteen known secondary metabolites. Their structures were established by extensive NMR spectroscopy and mass spectrometry analyses, as well as by comparison with literature data of their analogs.

## Introduction

Endophytic fungi are organisms that reside almost ubiquitously inside the fresh healthy tissue of plants, and they may increase the resistance of the host tropical trees to survive in extreme conditions [1]. As the global diversity of endophytic fungi is far from being accessed [2–3], they have been considered as an untapped microbial reservoir capable of producing a wide range of structurally unique natural products with potent pharmacological effects [4]. However, the production of biologically active compounds by filamentous fungi, especially those exclusive to their host plants, is not only important from a biomolecular standpoint but also from an ecological perspective. In continuation of our interest to explore secondary metabolites of rare and hitherto unexplored fungi hosted in Cameroonian medicinal plants [5–6], we investigated the chemical constituents of another new species of *Diaporthe*. This genus contains many plant pathogenic, endophytic, and saprobic species [7]. So far, the investigations of chemical constituents of *Diaporthe* species, have led to the isolation and characterization of a myriad of potent natural products with great structural variability such as polyketides, terpenoids, polyketide synthase–nonribosomal peptide synthetase (PKS–NRPS) alkaloids, and cytochalasins, which have been considered as taxonomic markers of the genus [7–10]. However, it is worthwhile to mention that the name *Phomopsis* should no longer be used since the introduction of the 1F1N concept in 2013, as explained by Chepkirui and Stadler [7]. Unfortunately, some authors who have been working on these fungi ever since the 1F1N concept was established in 2013 are apparently not aware of the fact *Diaporthe* is the older name and therefore takes preference over *Phomopsis*. Regarding the potent talents of *Diaporthe*, we are on the quest to the exploration of structure–activity relationships of cytochalasins to establish their trends for various medical applications [5]. Along the same lines, we herein report the isolation and structural elucidation of a new polyketide **1** and a new acetylated alternariol derivative **2** as well as fifteen known compounds including three alternariol derivatives **3**–**5**, one chromone **6**, one biphenyl **7**, seven cytochalasins **8**–**14**, two cytosporones **15** and **16**, and one macrolide **17** from the endophytic fungus, *Diaporthe cameroonensis*, isolated from the root of *Trema guineensis*, a Cameroonian medicinal plant from the family Ulmaceae [11].

## Results

### Structural elucidation

**Figure 1:**
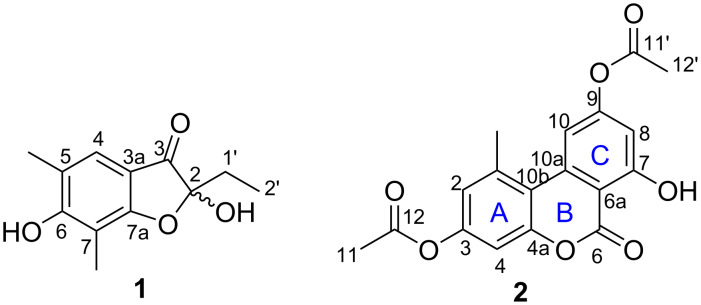
Chemical structures of compounds **1** and **2**.

*Diaporthe cameroonensis*, which showed an interesting high-performance liquid chromatography–diode array detector–high-resolution mass spectrometry (HPLC–DAD–HRMS) profile, was scale up fermented to give 12 g of culture extract. This extract was subjected to a silica gel column chromatography to give several fractions, which were further puriﬁed over normal and reversed-phase HPLC to yield 2-ethyl-2,6-dihydroxy-5,7-dimethylbenzofuran-3-one (**1**), 3,9-diacetylalternariol (**2**) (Figure 1), together with fifteen known compounds (Figure S1 in Supporting Information File 1).

**Figure 2:**
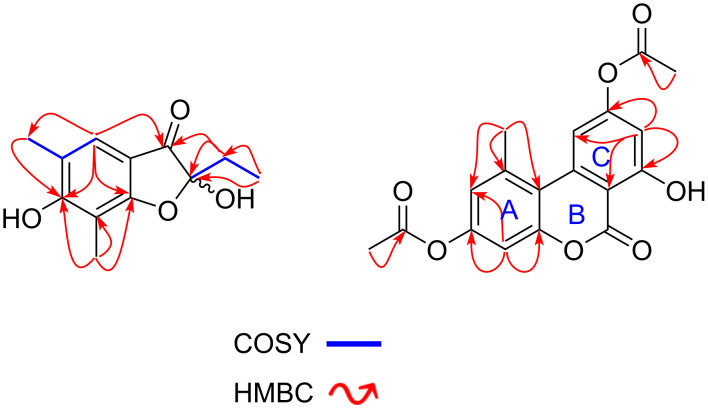
Key COSY and HMBC correlations of compounds **1** and **2**.

Compound **1** was obtained as a yellow oil. Its molecular formula, C_12_H_14_O_4_ that fits with six double bond equivalents, was established from the positive-ion mode HRESIMS (Figure S2, Supporting Information File 1), showing the pseudo-molecular ion peak [M + H]^+^ at *m*/*z* 223.0961 (calcd for C_12_H_15_O_4_, 223.0965). The ^1^H NMR spectrum (Figure S3 in Supporting Information File 1 and Table 1) displayed in the aromatic region, a singlet signal at δ_H_ 7.12 and in the shielded region, resonances for two aromatic methyl groups at δ_H_ 2.09 (s) and 2.02 (s). Characteristic signals at δ_H_ 1.75 (q, 7.0 Hz, 2H) and 0.75 (t, 7.0 Hz, 3H) indicated an ethyl group and were confirmed in the COSY spectrum with cross-peaks between the methylene and the methyl proton signals. The five aforementioned protons showed HSQC cross peaks with their respective carbon signals at δ_C_ 122.0, 28.8, 16.6, 8.1, and 7.1, respectively (Figure S5 in Supporting Information File 1). In the HMBC spectrum, key correlations were observed between the olefinic proton signal (δ_H_ 7.12) and six carbon signals including three deshielded ones at δ_C_ 196.1 for a ketone group, 169.2 and 165.6 attributed to oxygenated aromatic carbons, and three other signals at δ_C_ 109.4, 106.2, and 16.6 (Figure S6 in Supporting Information File 1). On the other hand, each of the two aromatic methyl signals displayed a set of three cross peaks; the first one at δ_H_ 2.09 with the carbon signals at δ_C_ 165.6, 122.0, and 120.3, and the second one at δ_H_ 2.02 with the carbon signals at δ_C_ 169.2, 165.6, and 106.2 (Figure 2). These findings clearly indicated the presence of the 1,3-dimethylbenzene moiety linked to a side chain containing an ethyl group and a ketone group. Furthermore, the ethyl group was attached to a hemiketal group (δ_C_ 106.1) as evidenced by the HMBC cross-peaks observed between the methylene proton signals at δ_H_ 1.75 with the carbon signals at δ_C_ 196.1, 106.1, and 7.1, and also between the methyl proton signals at δ_H_ 0.75 with the carbon signals at δ_C_ 106.1 and 28.8. Hence, these key correlations permitted to assign the attachment of the hemiketal carbon (δ_C_ 106.1) to the ketone group and the benzene ring. Based on the above evidence, the structure of **1** was assigned with a trivial name of 5,7-dimethylpseudopithonone (Figure 1). Pseudopithonone was isolated from the marine-derived fungus *Pseudopithomyces maydicus* [12]. The proposed structure was fully supported (Table 1) by HSQC-DEPT, HMBC, and COSY spectra (Figures S5–S7 in Supporting Information File 1). Key HMBC correlations of **1** are illustrated in Figure 2. This compound was found to be a racemate since no specific optical rotation was observed.

**Table 1:** ^1^H (500 MHz) and ^13^C (125 MHz) NMR data of compounds **1** and **2** in DMSO-*d*_6_.

no.	**1**		no.	**2**	
	
	δ_H_ (mult., *J* in Hz)	δ_C_ (mult.)		δ_H_ (*mult.*, *J* in Hz)	δ_C_ (mult.)

2	–	106.1 (C)	1	–	137.8 (C)
3	–	196.1 (C)	2	6.68 (d, 2.0)	117.0 (CH)
3a	–	109.4 (C)	3	–	153.2 (C)^a^
4	7.12 (s)	122.0 (CH)	4	6.55 (d, 2.0)	101.1 (CH)
5	–	120.3 (C)	4a	–	153.2 (C)^a^
6	–	165.6 (C)	6	–	158.5 (C)
7	–	106.2 (C)	6a	–	110.3 (C)
7a	–	169.2 (C)	7	–	156.9 (C)
1′	1.75 (q, 7.0)	28.8 (CH_2_)	8	6.62 (d, 1.0)	109.6 (CH)
2′	0.75 (t, 7.0)	7.1 (CH_3_)	9	–	154.2 (C)
5-CH_3_	2.09 (s)	16.6 (CH_3_)	10	7.57 (d, 1.0)	108.6 (CH)
7-CH_3_	2.02 (s)	8.1 (CH_3_)	10a	–	139.3 (C)
			10b	–	108.6 (C)
			1-CH_3_	2.71 (s)	25.2 (CH_3_)
			3-OAc, 9-OAc	2.28 (s)	21.0 (CH_3_) 169.0 (C)
			7-OH	10.33 (s)	–

^a^Overlapped signals.

3,9-Diacetylalternariol (**2**, Figure 1) was isolated as a white amorphous powder and was assigned the molecular formula C_18_H_14_O_7_ (12 DBE), as deduced from the pseudo-molecular ion peak [M + H]^+^ at *m*/*z* 343.0809 (calcd for C_18_H_15_O_7_, 343.0812) observed in the (+)-HRESIMS spectrum (Figure S8 in Supporting Information File 1). Its ^1^H NMR spectrum (Figure S9, Supporting Information File 1) allowed the deduction of an alternariol scaffold [13] with signals of a chelated hydroxy proton at δ_H_ 10.33, two pairs of *meta*-coupled protons at δ_H_ 6.68 and 6.55 (d, 2.0 Hz, 1H each), and at δ_H_ 7.57 and 6.62 (d, 1.0 Hz, 1H each), in addition to a singlet signal at δ_H_ 2.71 (s, CH_3_, 3H). The alternariol skeleton was further confirmed by the ^13^C NMR and HSQC spectra (Figures S10 and S11 in Supporting Information File 1), in conjunction with the HMBC spectrum (Figure S12 in Supporting Information File 1) which showed cross-peaks from the proton signal at δ_H_ 6.62 (H-8) to the carbon signals at δ_C_ 156.9 (C-7), 154.2 (C-9), 110.3 (C-6a), and 108.6 (C-10); and also between the proton signal at δ_H_ 6.55 (H-4) and the carbon signals at δ_C_ 153.2 (C-3, C-4a), 117.0 (C-2), and 108.6 (C-10b) (Figure 2 and Table 1). Further HMBC correlations (Figure 2) were observed between the proton signal of the methyl group at δ_H_ 2.71 with the carbon resonances δ_C_ 137.8 (C-1), 117.0 (C-2), and 108.6 (C-10b). Additionally, the ^1^H NMR spectrum displayed two singlet signals overlapping at δ_H_ 2.28 due to two acetyl groups, which showed strong HMBC cross peaks with the carbonyl carbon signals at δ_H_ 169.0. The structure of **2** was fully assigned by using the ^13^C NMR spectrum which displayed sixteen carbon signals sorted into three methyl signals of which two overlapping ones at δ_C_ 21.0 and one at δ_C_ 25.2 attached to the alternariol basis skeleton (Figure 2, Table 1); four methines (δ_C_ 117.0, 101.1, 109.6, 108.6), and eleven quaternary carbons including the signal of the lactone carbon at δ_C_ 158.5 (C-6) and those of the three sp^2^ oxygenated carbons at δ_C_ 156.9, 154.2, and 153.2. Key COSY and HMBC correlations for **2** are illustrated in Figure 2 and the complete assignment for all protons and carbons is tabulated in Table 1. As we have been able to detect the compound by HPLC–MS in methanolic crude extracts of the fungus that did not come into contact with ethyl acetate or acetic acid (data not shown), we can exclude that it is an isolation artefact.

The fifteen known compounds isolated from *Diaporthe cameroonensis* extract, which included alternariol (**3**) [14], 2-hydroxyalternariol (**4**) [15], 4-hydroxyalternariol (**5**) [16–17], 2,5-dimethyl-7-hydroxychromone (**6**) [18], decarboxyaltenusin (**7**) [19], cytochalasin H (**8**) [20], epoxycytochalasin H (**9**) [21], cytochalasin J (**10**) [22], cytochalasin J2 (**11**) [23], cytochalasin J3 (**12**) [23], cytochalasin N (**13**) [24], cytochalasin RKS-1778 (**14**) [25], cytosporone C (**15**) [26], cytosporone E (**16**) [27], and lasiodiplodin (**17**) [28], were identified based the comparison of their spectral data with the previously published ones.

## Discussion

The present study describes the isolation of two previously undescribed polyketides, named 5,7-dimethylpseudopithonone (**1**) and 3,9-diacetylalternariol (**2**) along with cytochalasins and cytosporones from *Diaporthe cameroonensis*. The presence of cytochalasins and cytosporones was obvious, as they constitute the taxonomic markers of the genus *Diaporthe* [8]. Other classes of compounds including chromones, chromanones, benzofuranones, and macrolides reported in this study, were also isolated from the genus *Diaporthe* [8,26].

Compound **1** is a benzofuranone, structurally related to enalin A and pseudopithonone, isolated from the fungus *Verruculina enalia* and the marine-derived fungus *Pseudopithomyces maydicus*, respectively [12,29]. Its core structure is also like actiketal, a new member of the glutarimide antibiotics, previously isolated from *Streptomyces* [30] and whose antimicrobial activity is probably related to the glutarimide moiety. In compound **1**, the lack of this moiety, in addition to the fact that it has been isolated as a racemate could not lead to any beneficial property.

As for compound **2**, it is a diacetylated derivative of alternariol, a heptaketide coumarin derivative with a fused tricyclic ring called dibenzo-α-pyrone [31–32]. Alternariol is one of the toxic metabolites isolated from *Alternaria* strains, that grow on various natural resources such as corn, rice, fruits, vegetables, oilseeds, juices, wins, and cereals [31]. The isolation of the alternariol compound in this study is not surprising because it has been reported that dibenzo-α-pyrones are also found in mycobionts, plants, some animal dung, and endophytes [32–33]. Although the toxicity of dibenzo-α-pyrones is not fully understood and varies amongst cellular systems [31], alternariols have been identified in various bioassay systems as toxic compounds [34]. Furthermore, the estrogenic potential of alternariols and their inhibitory effects on cell proliferation have been demonstrated [35], and several other beneficial bioactivities of dibenzo-α-pyrones have been reported [32]. The importance of dibenzo-α-pyrones is manifold as they can also be used as key intermediates in the synthesis of therapeutic compounds [32]. Since compound **1** was isolated as a racemic mixture, while compound **2** is a diacetylated derivative of the mycotoxin alternariol known for its mutagenic properties, no further beneficial effects could be expected with them. However, the isolation of these compounds further expands the understanding of the metabolic diversity of *Diaporthe*.

## Conclusion

The study of the new fungal strain, *Diaporthe cameroonensis* isolated from a Cameroonian medicinal plant, *Trema guineensis* led to the isolation of seventeen secondary metabolites, including two previously undescribed polyketides, namely 5,7-dimethylpseudopithonone (**1**) and 3,9-diacetylalternariol (**2**). This study has contributed further to our knowledge of the metabolic diversity within *Diaporthe* and its meanwhile invalid synonymic anamorphic state *Phomposis*. Likewise, the other, known co-metabolites like the cytochalasins that were concurrently obtained are presently being studied in-depth by methods of cell biology. However, this activity is beyond the scope of the current study.

## Experimental

### General experimental procedures

Column chromatography (60.4 cm length × 5.5 cm inner diameter) was carried out on silica gel 230–400 mesh (Merck). Analytical TLC was performed on Merck pre-coated silica gel 60 F_254_ plates, and spots were detected using ceric sulfate spray reagent and/or diluted sulfuric acid before heating. LC–MS chromatograms were recorded with an UltiMate 3000 Series uHPLC (Thermo Fischer Scientific) using a C18 Acquity UPLC BEH column (2.1 × 50 mm, 1.7 µm) connected to an amazon speed ESI-Iontrap-MS (Bruker).

Semi-preparative and/or preparative HPLC systems on normal and reversed phases (RP) were used for the purification of compounds. RP flash and p-HPLC systems (PLC 2050, Gilson, Wisconsin-USA; and Flash and prep HPLC, C-850, Büchi) were equipped with appropriate columns viz., VP Nucleodur C18 HTec (10 µm, 250 × 40 mm, Macherey-Nagel, Germany), Gemini C18 (10 µm, 250 × 50 mm, Phenomenex), and Nucleosil 120 OH Diol (7 µm, 250 × 21 mm, Machery-Nagel, Düren, Germany) columns maintained at room temperature. Normal phase p-HPLC equipped with DAD detector (Agilent 1100 Series, Santa Clara, USA) was connected to a Nucleosil 120 OH Diol column.

Deionized water used for RP p-HPLC was obtained from a Milli-Q water purification system (Millipore, Schwalbach, Germany) and all organic solvents and reagents were of analytical grade. Evaporation of solvents from fractions was performed using rotary evaporators equipped with a vacuum controller and diaphragm pump vacuum (Hei-VAP Expert, Germany).

HRESIMS spectra were recorded with an Agilent 1200 Infinity Series HPLC-UV system (Agilent Technologies; column 2.1 × 50 mm, 1.7 µm, C18 Acquity UPLC BEH (waters), solvent A: H_2_O + 0.1% formic acid; solvent B: ACN + 0.1% formic acid, gradient: 5% B for 0.5 min increasing to 100% B in 19.5 min and then maintaining 100% B for 5 min, flow rate 0.6 mL/min, UV–vis detection 200–640 nm) connected to a MaXis ESI-TOF mass spectrometer (Bruker) (scan range 100–2500 *m*/*z*, capillary voltage 4500 V, dry temperature 200 °C).

NMR spectra were recorded at 25 °C on a Bruker (Billerica, MA/USA) 500 MHz Avance III spectrometer with a BBFO (plus) Smart Probe (^1^H NMR: 500 MHz and ^13^C NMR: 125 MHz). Chemical shifts (δ) were reported in ppm using tetramethylsilane (TMS) (Sigma-Aldrich) as an internal standard, while coupling constants (*J*) were measured in hertz (Hz).

Optical rotations were measured in methanol by using an Anton Paar MCP-150 polarimeter (Seelze, Germany) at 20 °C.

### Isolation and identification of the endophytic fungus

The fresh root of *Trema guineesis* was collected in January 2019 in Kala Mountain (near Yaoundé), in the Centre region of Cameroon. It was identified at the National Herbarium of Cameroon, Yaoundé, where a voucher specimen was deposited under the number 42166/HNC. The isolation of fungi from the plant material was carried out following the previously described methodology [6]. The fungus was recently introduced as the new species *Diaporthe cameroonensis* by Lambert et al. [36]. The strain was deposited in the fungarium of the Helmholtz Centre for Infection Research (HZI, Braunschweig, Germany) under the reference number STMA 18289.

### Fermentation, extraction and isolation

Well-grown agar plate cultures of the *Diaporthe cameroonensis* were used to inoculate 45 Erlenmeyer flasks of 500 mL, each containing 80 g of rice and 100 mL distilled water and incubated at 25 °C. After 28 days, the cultures were harvested. The fungal mycelia were further extracted with ethyl acetate and the solution was concentrated under reduced pressure to yield 12.2 g of crude extract. 12 g of this extract was subjected to a silica gel (230–400 mesh) column chromatography using a stepwise gradient of CH_2_Cl_2_/MeOH (ranging from 0 to 100% of MeOH, v/v), to afford a total of 8 main series (A–H) obtained on the basis of TLC analyses. However, series A (2.5 g) obtained with pure CH_2_Cl_2_ and series H (2.3 g) eluted with CH_2_Cl_2_/MeOH 85:15 (v/v) were found to be complex mixtures and were not further investigated. The guided LC–MS purifications of the other series (B–G) were achieved by using semi-preparative and preparative HPLC systems on normal and reversed phases.

Series B (462 mg) obtained with CH_2_Cl_2_/MeOH 97.5:2.5 (v/v) was subjected to a flash chromatography system (PLC 2020, Gilson) equipped with a VP Nucleodur C18 HTec column (10 µm, 250 × 40 mm). The chromatographic separation was performed by using a constant flow rate of 10 mL/min with the mobile phases made with solvent A: deionized water (H_2_O) + 0.1% formic acid (FA) and solvent B: acetonitrile (ACN) + 0.1% formic acid. The binary gradient was: linear of 5% B for 5 min, automatic stepwise gradient from 5 to 100% B for 5–105 min, followed by 100% B for 10 min wash, and finally by 80% ACN + 20% H_2_O for 10 min re-equilibration time. Peaks were detected by a diode array detector in the range of 210–600 nm and were automatically collected into glass tubes. Out of the hundred and twenty-seven tubes, twenty (20) tubes resulting from UV peaks were selected including peaks of compounds **2** (1.18 mg; *t*_R_ = 52 min), and **12** (2.93 mg, white neat solid, *t*_R_ = 85 min). The LC–MS analysis of other fractions suggested the presence of very low amounts of compounds and these fractions were not further investigated.

Series C (1.2 g) eluted with CH_2_Cl_2_/MeOH 97.5:2.5 (v/v) was divided into three portions of 400 mg each. Every portion was purified on a flash liquid chromatography (Flash and prep HPLC, C-850, UV/ELSD detector, UV–vis/UV scanning) equipped with a Gemini C18 (10 µm, 250 × 50 mm). Equivalent fractions from the three repeated runs were combined to give compounds **6** (1.19 mg, white solid, *t*_R_ = 4.86 min), **8** (286 mg, white crystal, *t*_R_ = 8.31 min), **13** (1.42 mg, white crystal, *t*_R_ = 9.42 min).

Series D (682 mg) obtained by a silica gel column chromatography with CH_2_Cl_2_/MeOH 95:5 (v/v) was divided into two portions of 341 mg each. After dissolving every portion, injections were also achieved on a preparative HPLC system. Equivalent fractions from the three repeated runs were combined to give three sub-fractions (D_1_–D_3_). All these sub-fractions D_1_ (12 mg), D_2_ (8 mg), and D_3_ (15.8 mg) were further separately purified over normal phase preparative HPLC with a DAD detector with a Nucleosil 120 OH Diol column (7 µm, 250 × 21 mm) used as the stationary phase and the solvent mixture CH_2_CH_2_/MeOH 92:8 (v/v) as the mobile phase. The elution was performed for 25 min each with a flow rate of 2 mL/min, an automatic pressure in the range of 1–10 bar and the UV absorptions were set at 210, 254, and 366 nm to yield compounds **15** (3.2 mg, yellow oil, *t*_R_ = 8 min), **9** (1.3 mg, yellow oil, *t*_R_ = 14 min), **3** (2.76 mg, white neat solid, *t*_R_ = 16 min), and **1** (0.79 mg, yellow oil, *t*_R_ = 22 min), respectively.

Series E (294 mg) was further purified to yield compounds **5** (0.95 mg, white amorphous powder, *t*_R_ = 35.20 min), **17** (6.1 mg, white amorphous powder, *t*_R_ = 11.21 min), and **7** (1.45 mg, yellow neat solid, *t*_R_ = 15.00 min), **4** (2.45 mg, white amorphous powder, *t*_R_ = 22.00 min), respectively. Successive purifications of series F (898 mg) over normal (CH_2_CH_2_/MeOH 92:8, Nucleosil 120 OH Diol column) and reversed-phase preparative HLPC (Gilson, PLC 2020, solvents A/B in 0.1% FA: H_2_O/ACN) gave compound **16** (1.31 mg, yellow neat solid, *t*_R_ = 30.22 min). Series G (578 mg) obtained with CH_2_Cl_2_/MeOH 88:12 (v/v) was also purified to give compounds **14** (5.93 mg, white oil, *t*_R_ = 35.20 min), **11** (8.33 mg, white oil, *t*_R_ = 38.17 min), **10** (1.60 mg, yellow oil, *t*_R_ = 45.12 min), and **15** (1.23 mg, white oil).

**5,7-Dimethylpseudopithonone (1):** yellow oil, 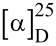 0 (*c* 0.27, MeOH); UV (MeOH): λ_max_ (PDA): 218, 290, 342 nm; ^1^H NMR (500 MHz, CD_3_OD) and ^13^C NMR (125 MHz, DMSO-*d*_6_) are shown in Table 1; (+)-HRESIMS (*m*/*z*): [M + H]^+^ calcd for C_12_H_15_O_4_, 223.0965; found, 223.0961.

**3,9-Diacetylalternariol (2):** white amorphous powder, UV (MeOH): λ_max_ (PDA): 222, 258, 330 nm; ^1^H NMR (500 MHz, DMSO-*d*_6_) and ^13^C NMR (125 MHz, DMSO-*d*_6_) are shown in Table 1; (+)-HRESIMS (*m/z*): [M + H]^+^ calcd for C_18_H_15_O_7_, 343.0812; found, 343.0809.

## Supporting Information

File 1 HRESIMS data and ^1^H, ^13^C, COSY, HSQC, and HMBC NMR spectra of compounds **1** and **2**.
