# Supplementary material for: Secondary metabolites of Diaporthe cameroonensis, isolated from the Cameroonian medicinal plant Trema guineensis
Source: Beilstein J Org Chem. 2023 Oct 13;19:1555–61. doi: 10.3762/bjoc.19.112 (PMC10616697; doi:10.3762/bjoc.19.112)
Supplement: File 1 — HRESIMS data and 1H, 13C, COSY, HSQC, and HMBC NMR spectra of compounds 1 and 2. [file Beilstein_J_Org_Chem-19-1555-s001.pdf]

BEILSTEIN JOURNAL OF ORGANIC CHEMISTRY

Supporting Information

for

Secondary metabolites of *Diaporthe cameroonensis*, isolated from the Cameroonian medicinal plant *Trema guineensis*

Bel Youssouf G. Mountessou, Élodie Gisèle M. Anoumedem, Blondelle M. Kemkuignou, Yasmina Marin-Felix, Frank Surup, Marc Stadler and Simeon F. Kouam

Beilstein J. Org. Chem. **2023,** *19,* 1555–1561. doi:10.3762/bjoc.19.112

HRESIMS data and ^{1}H, ^{13}C, COSY, HSQC, and HMBC NMR spectra of compounds 1 and 2

License and Terms: This is a supporting information file under the terms of the Creative Commons Attribution License (https://creativecommons.org/licenses/by/4.0). Please note that the reuse, redistribution and reproduction in particular requires that the author(s) and source are credited and that individual graphics may be subject to special legal provisions.
The license is subject to the *Beilstein Journal of Organic Chemistry* terms and conditions: (https://www.beilstein-journals.org/bjoc/terms)

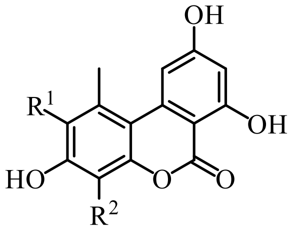

$R^1 = R^2 = H$	**3**
$R^1 = OH; R^2 = H$	**4**
$R^1 = H; R^2 = OH$	**5**

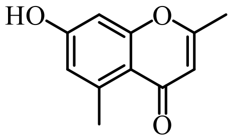

6

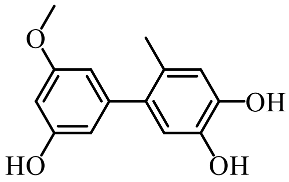

7

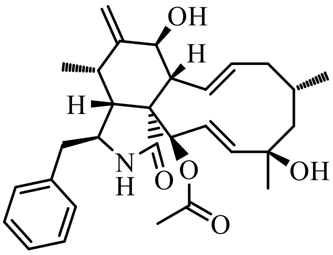

8

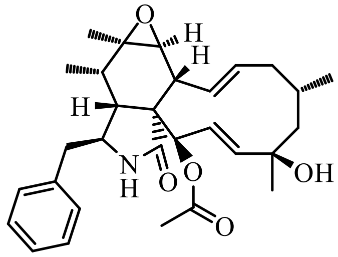

9

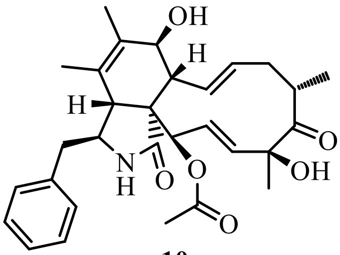

10

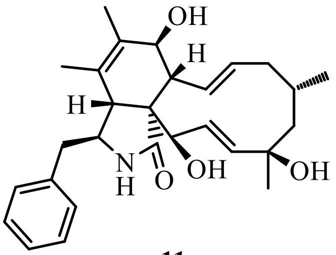

11

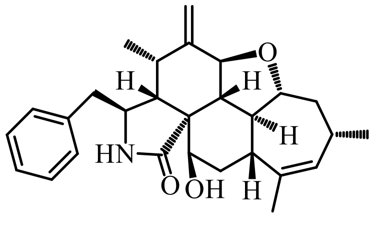

12

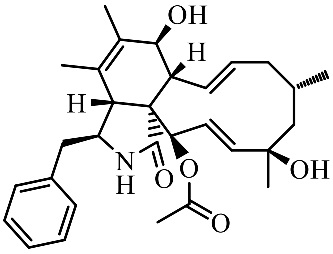

13

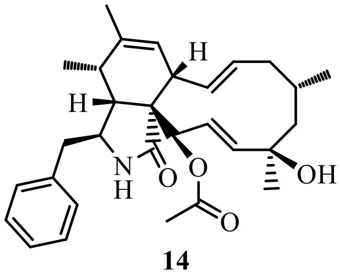

14

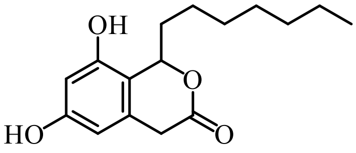

15

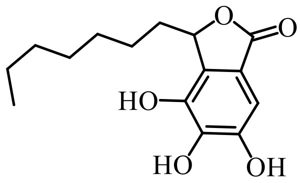

16

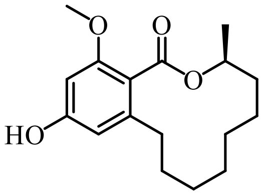

17

Figure S1: Chemical structures of compounds **3**–**17**.

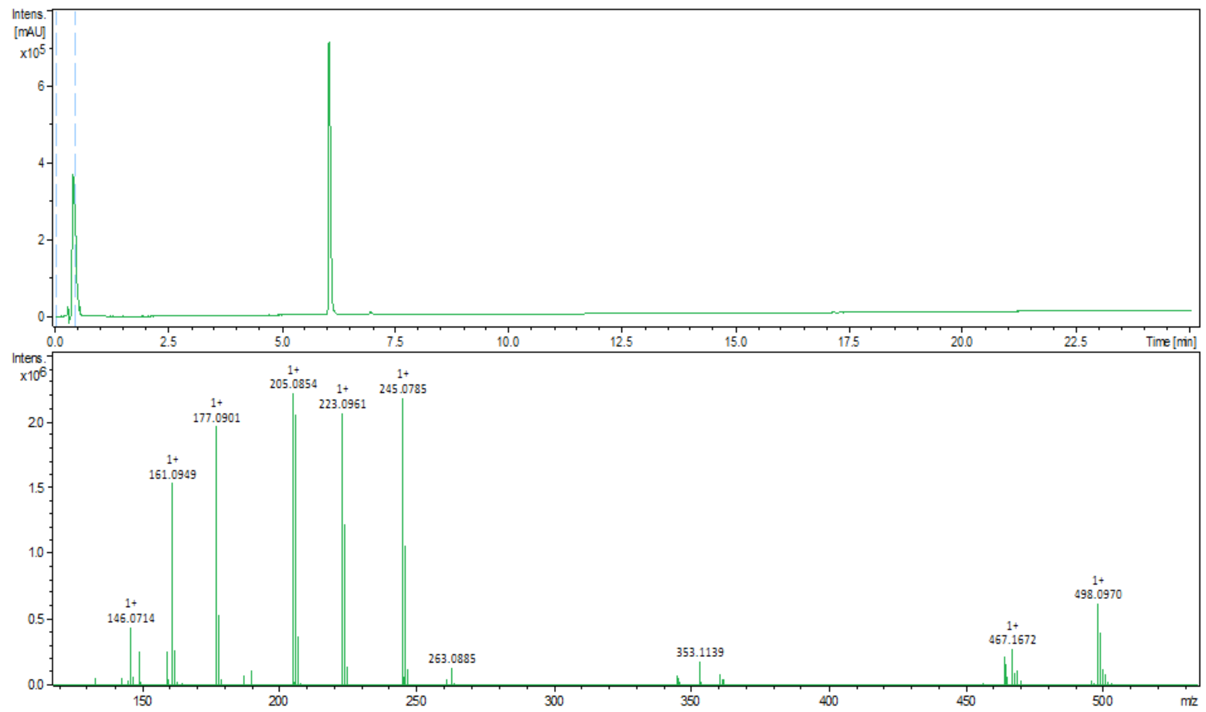

Figure S2: HPLC chromatogram (a) and positive-ion mode HRESIMS spectrum (b) of compound **1**.

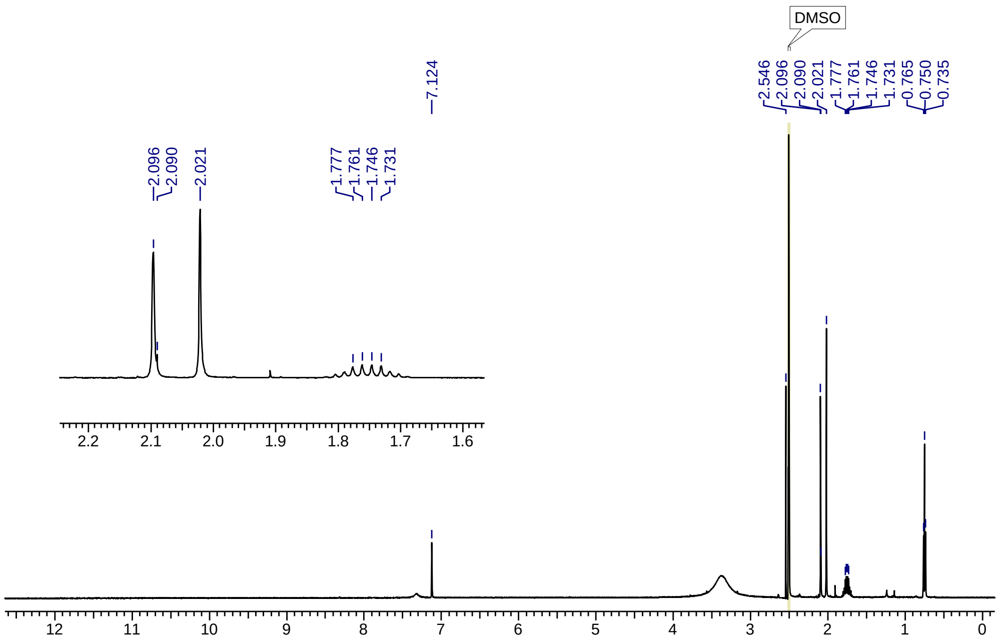

Figure S3: ^{1}H NMR spectrum of compound **1** (500 MHz, DMSO-d_6).

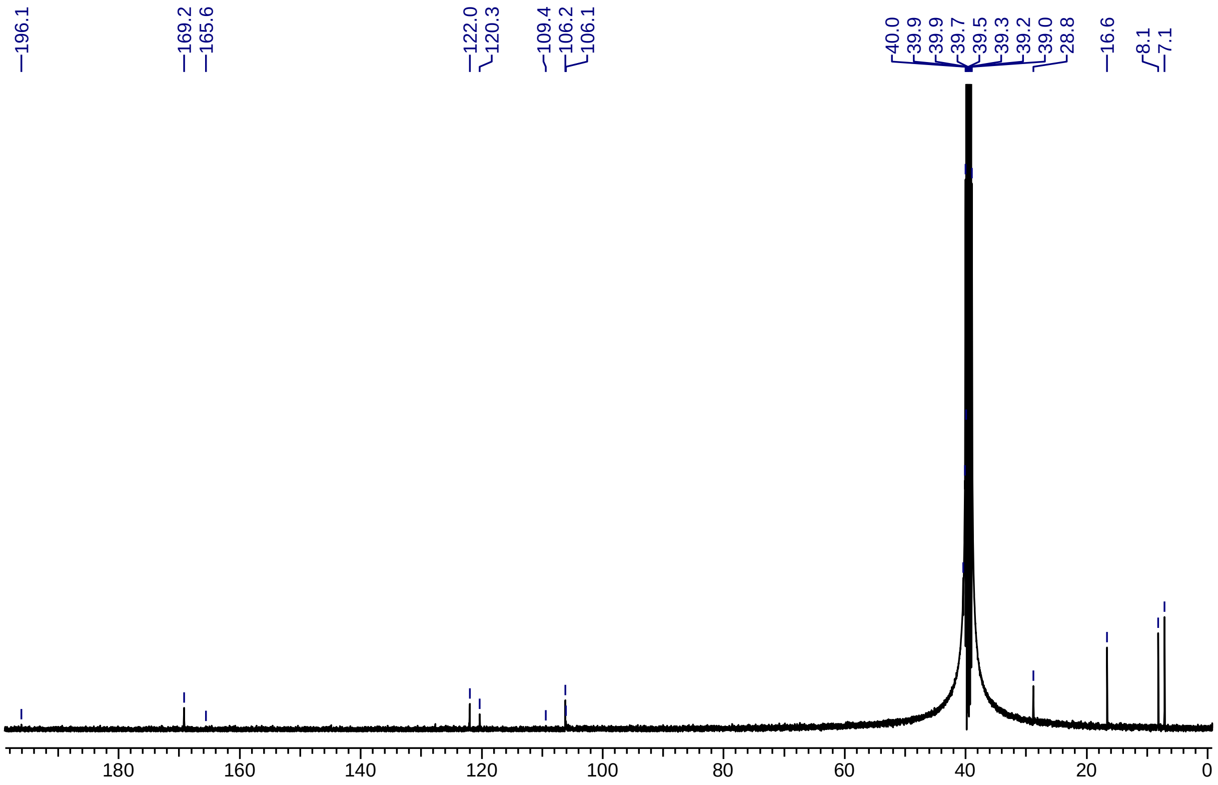

Figure S4: ^{13}C NMR spectrum of compound **1** (125 MHz, DMSO-d_6).

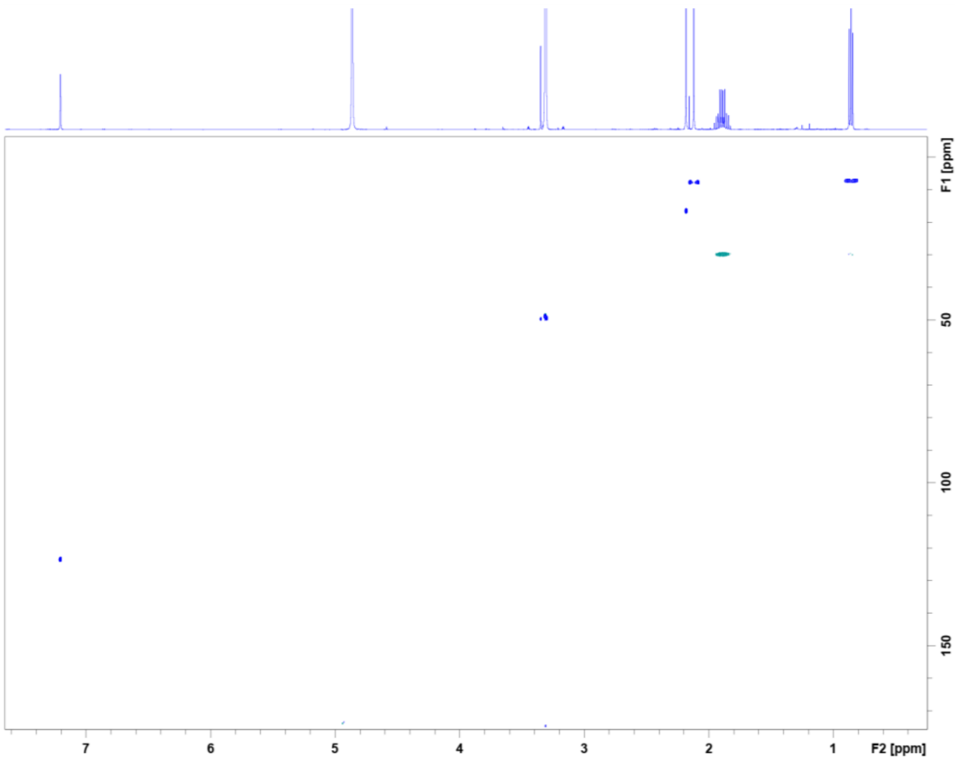

Figure S5: HSQC-DEPT spectrum of compound **1**.

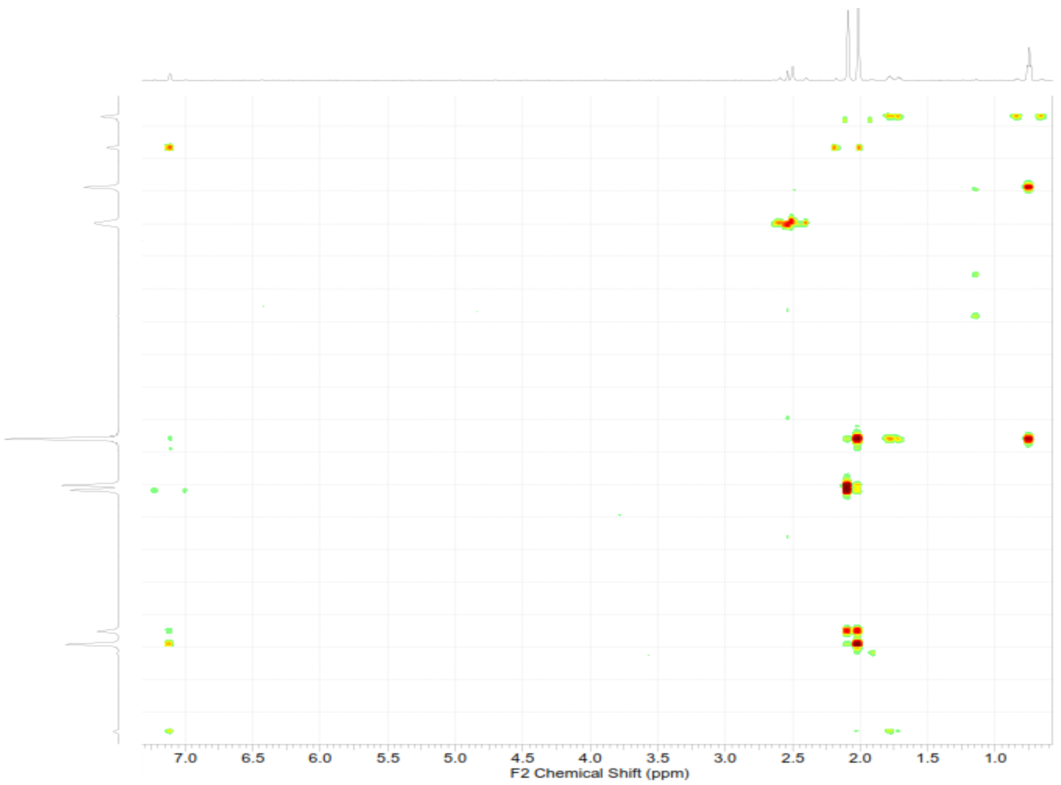

Figure S6: HMBC spectrum of compound **1**.

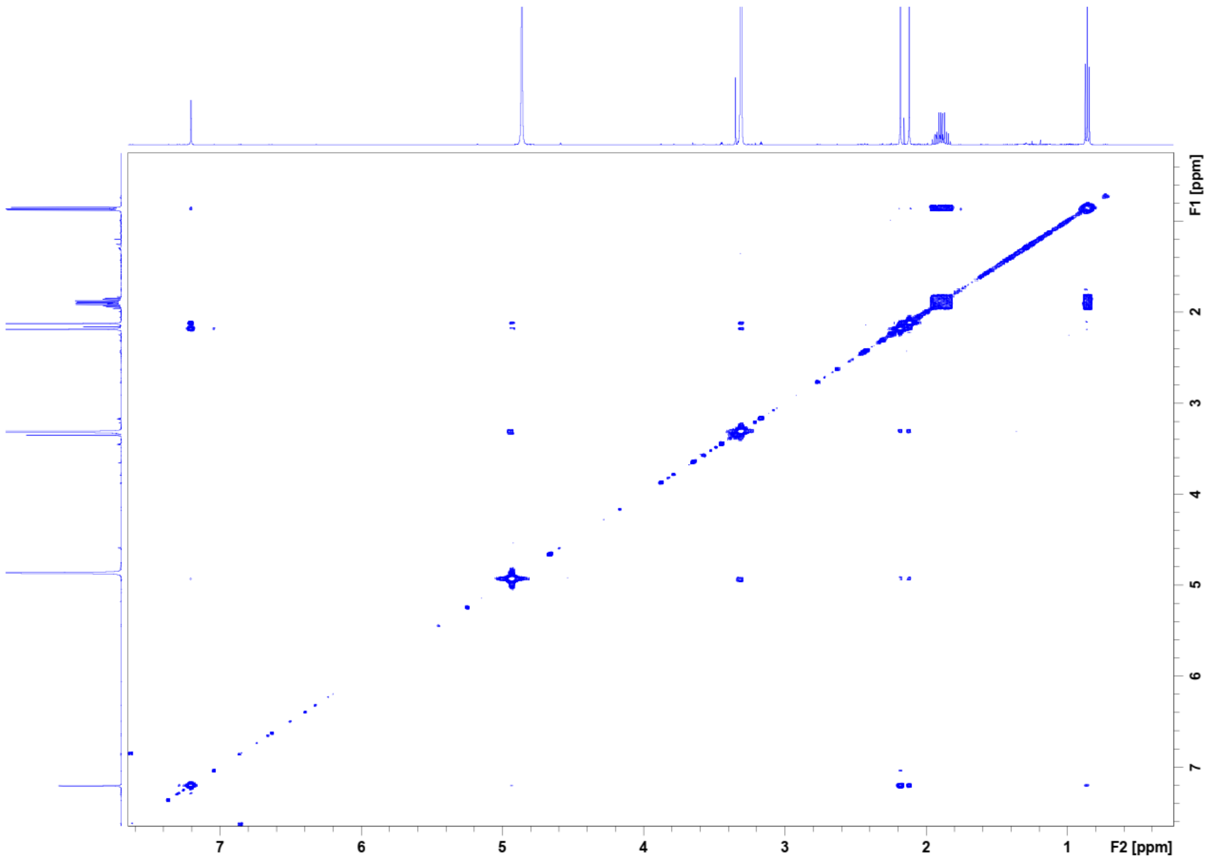

Figure S7: COSY spectrum of compound **1**.

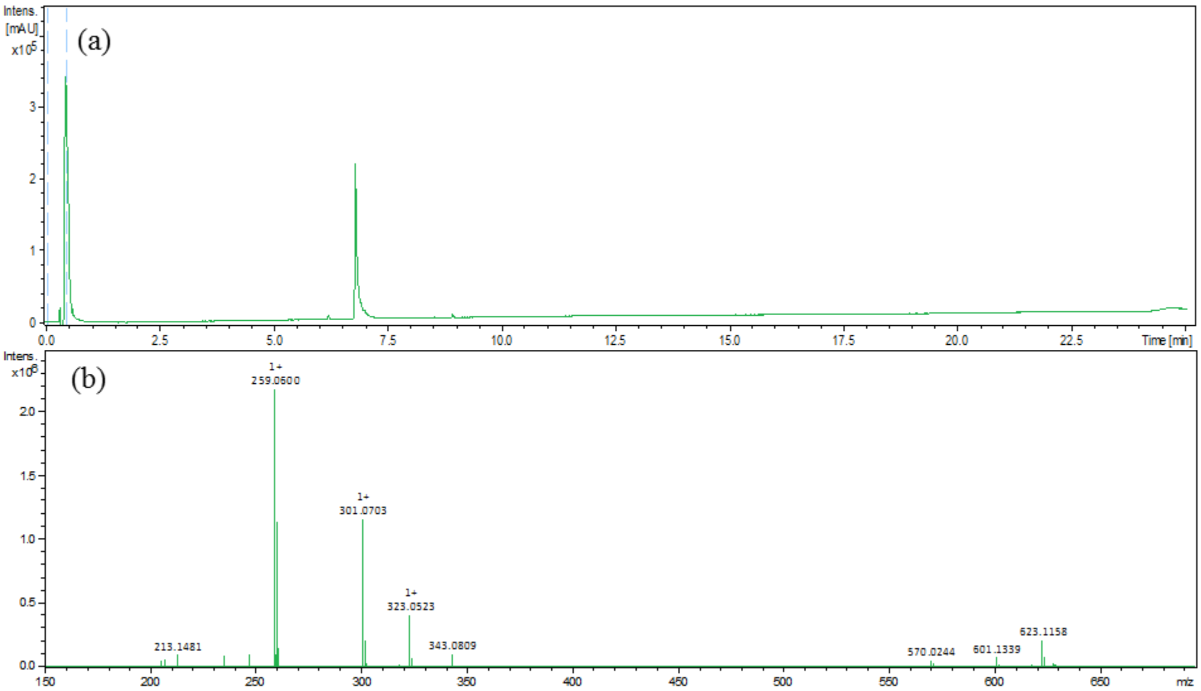

Figure S8: HPLC chromatogram (a) and positive ion mode HRESIMS spectrum (b) of compound **2**.

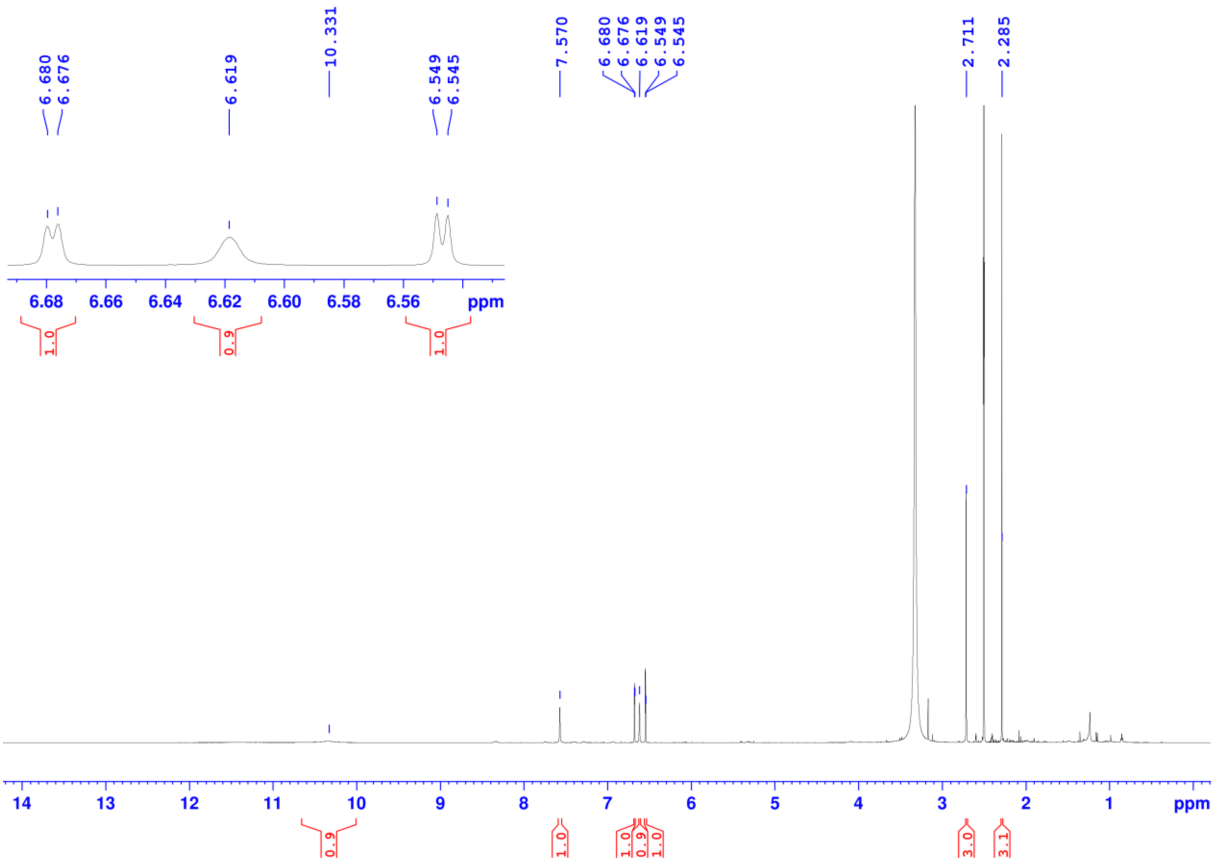

Figure S9: ^{1}H NMR spectrum of compound **2** (500 MHz, DMSO-d_6).

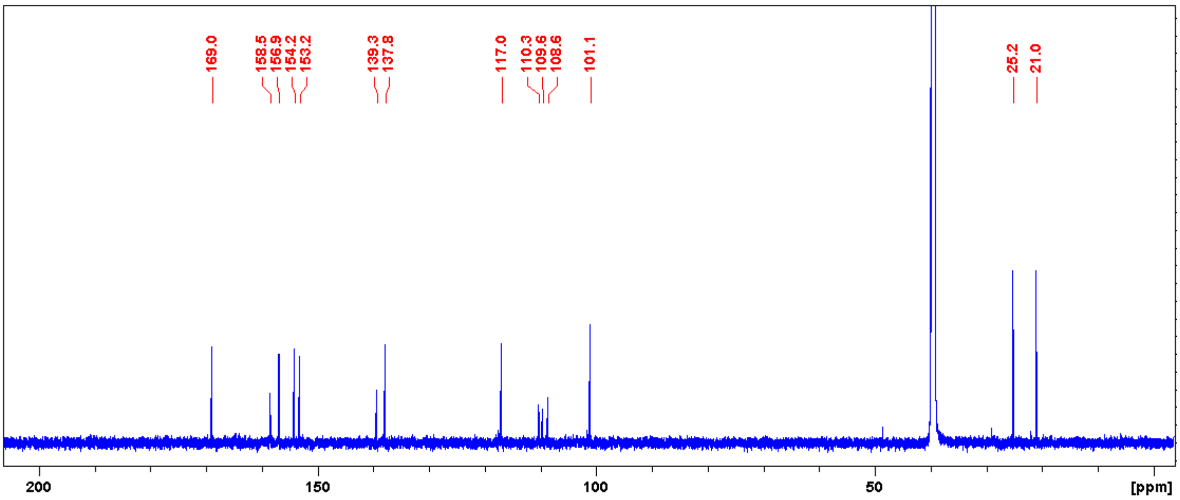

Figure S10: ^{13}C NMR spectrum of compound **2** (125 MHz, DMSO-d_6).

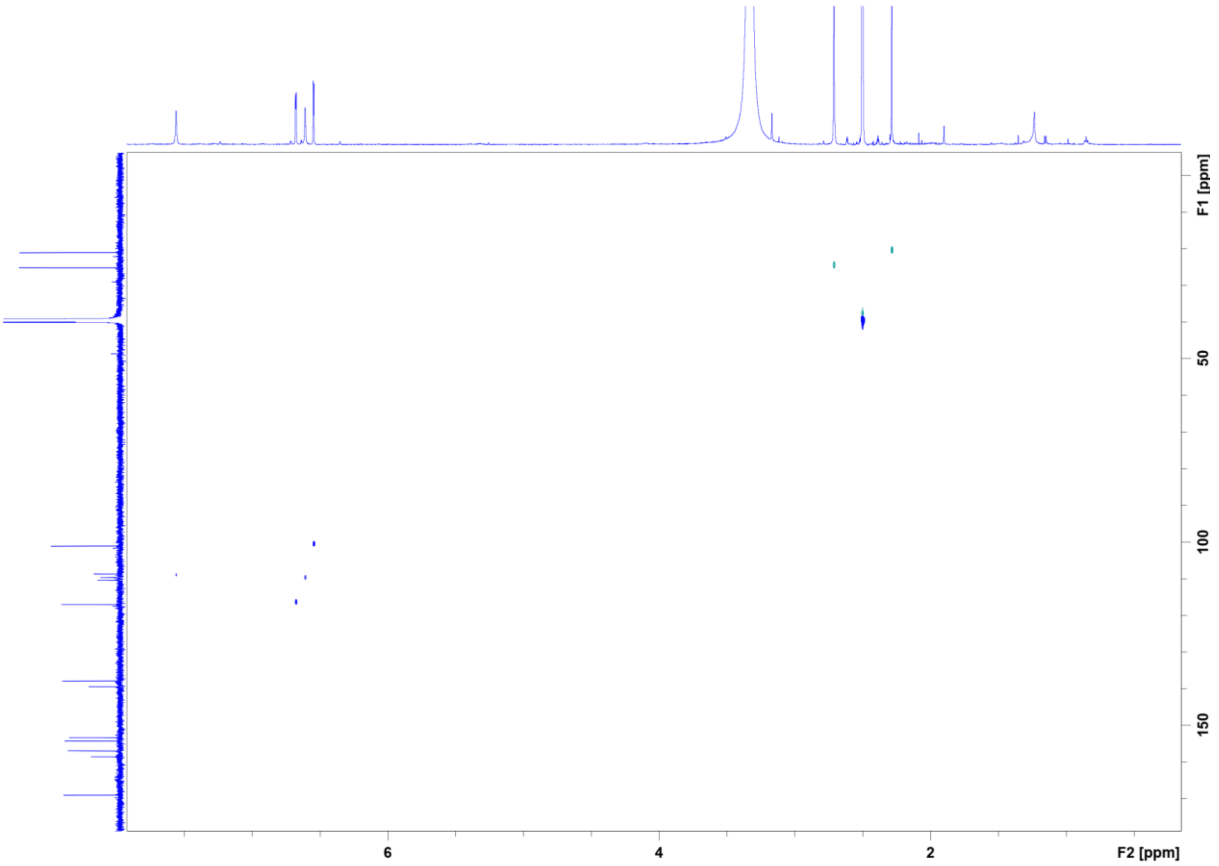

Figure S11: HSQC-DEPT spectrum of compound **2**.

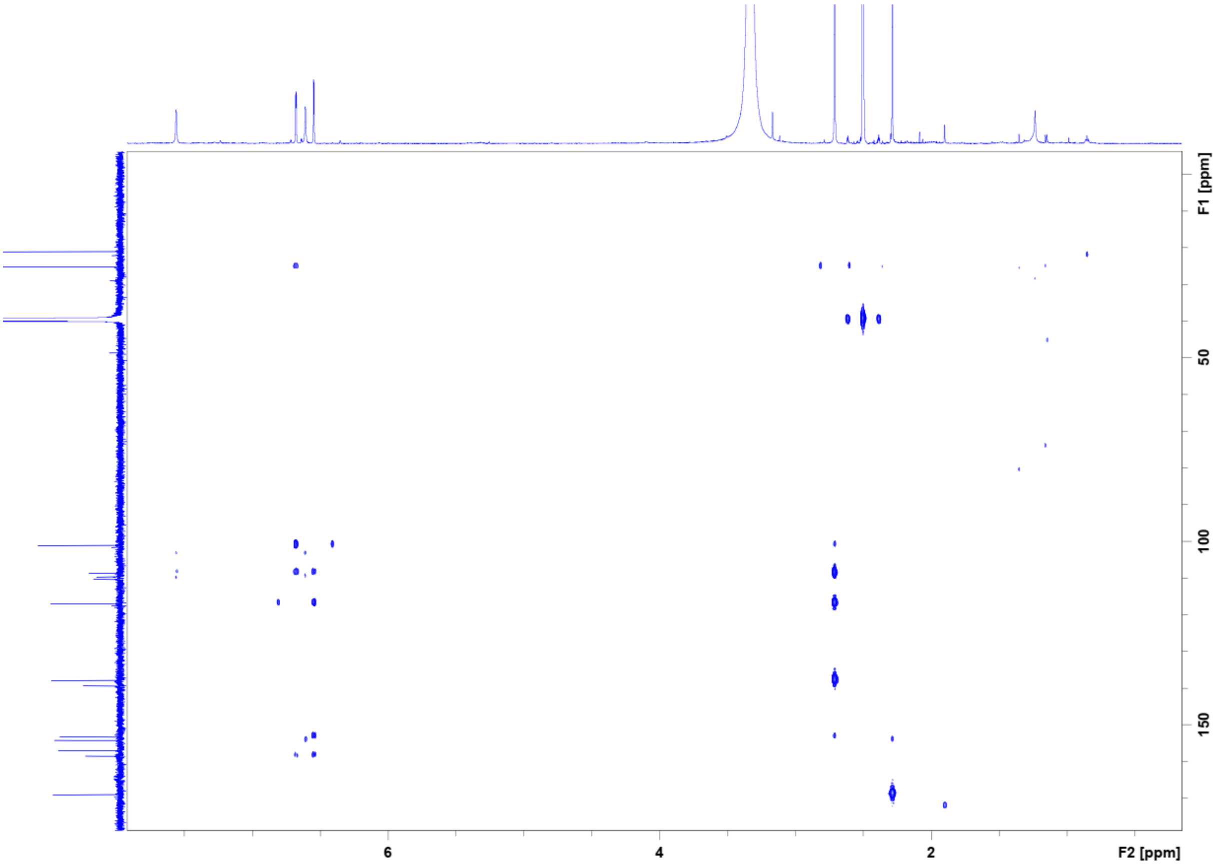

Figure S12: HMBC spectrum of compound **2**.

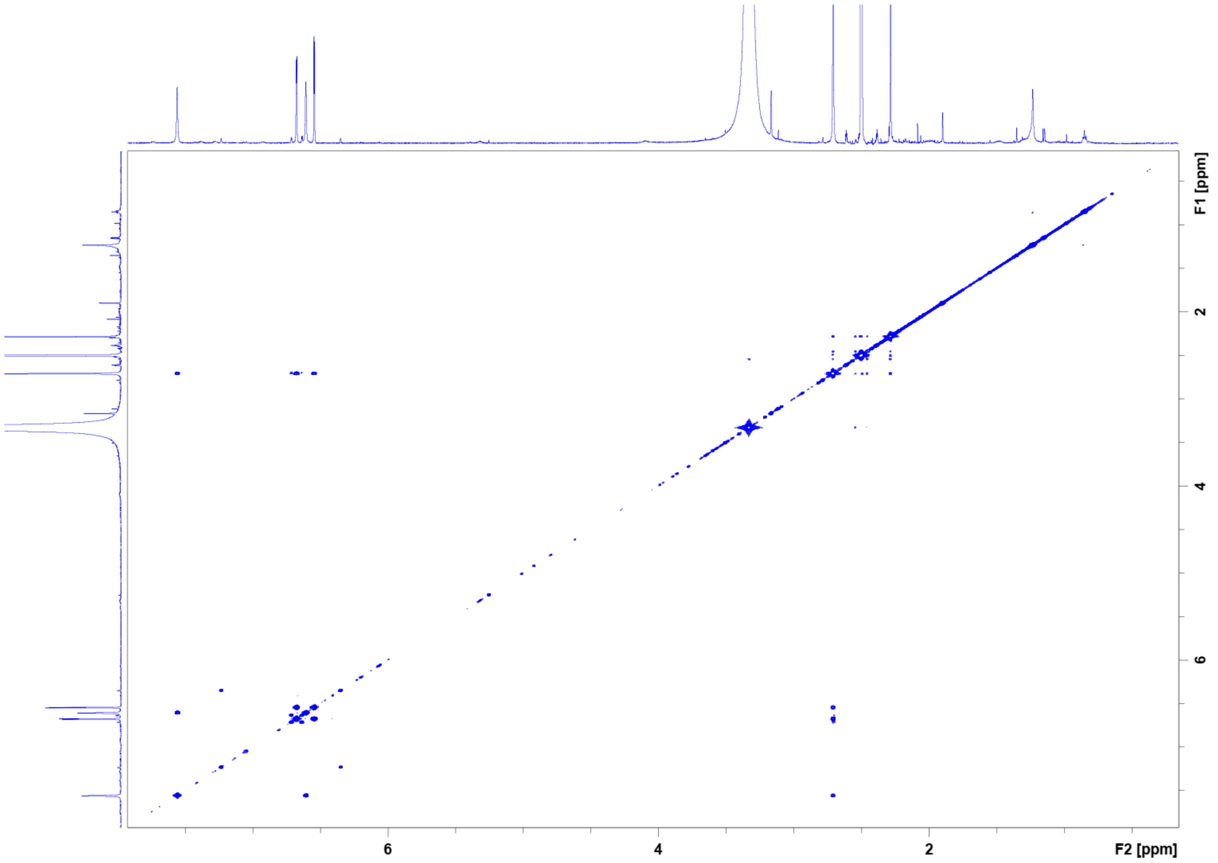

Figure S13: COSY spectrum of compound **2**.
